# Psychological impact of an epidemic on the mental health of medical staff: a case study of mobile cabin hospitals in Beijing during the COVID-19 pandemic

**DOI:** 10.3389/fpubh.2026.1746800

**Published:** 2026-04-09

**Authors:** Yu Tong Ni, Jing Fei Wang, Mi Na Wang, Lin Bo Shen, Qing Dai Li, Lin Liang, Jie Sun, Ning An Xiao, Jian Hao Liu, Xin Du, Jing Wang, Yuan Bo Fu

**Affiliations:** 1 Beijing Key Laboratory of Acupuncture Neuromodulation, The Department of Acupuncture and Moxibustion, Beijing Hospital of Traditional Chinese Medicine, Capital Medical University, Beijing, China; 2 Beijing University of Chinese Medicine, Beijing, China; 3 Beijing Xiao Tang Shan Hospital, Beijing, China; 4 Beijing Huairou District Hospital of Traditional Chinese Medicine, Beijing, China; 5 Sanya Traditional Chinese Medicine Hospital, Sanya, China; 6 Dongzhimen Hospital, Beijing University of Chinese Medicine, Beijing, China

**Keywords:** epidemic, GHQ-12, medical staff, mobile cabin hospitals, psychological status

## Abstract

**Purpose:**

To explore the impact of epidemic diseases outbreak on the psychological status of medical workers.

**Methods:**

A total of 266 online questionnaires were distributed to medical staff in several mobile cabin hospitals in Beijing. The questionnaires were divided into a General Demographic Characteristics Questionnaire and the 12-item General Health Questionnaire (GHQ-12), and a total score of ≥4 in the GHQ-12 was considered to be a decline in mental health.

**Results:**

A total of 266 members of medical staff participated in this study, and 30.1% experienced a decline in mental health recently. The highest prevalence of emotional distress was found among those who were female, married without children, working as doctors and other occupations, aged 30–39 years, working in the confirmation department, supporting the mobile cabin hospitals three times or more, the duration of this support for 1 month or longer, and having a previous state of anxiety and depression. In addition, females experienced higher rates of insomnia due to worry compared to males, as did medical staff in the confirmation department compared to the administration and logistics departments. In the same stressful environment, men and doctors had a greater rate of perceived loss of self-worth than women and nurses.

**Conclusion:**

The outbreak of epidemic makes medical workers face the risk of emotional distress. To achieve the purpose of preventing and treating emotional distress of medical workers, we need to carry out continuous and strict research and evaluation on the mental health planning of medical workers, formulate relevant policies and carry out professional psychological intervention.

## 1 Introduction

### 1.1 Background

Epidemic diseases are characterized by high infectivity, high pathogenicity and high mortality (1). And Coronavirus disease 2019 (COVID-19) is a kind of epidemic. since the World Health Organization (WHO) declared a global pandemic of COVID-19 on March 11, 2020, it has brought severe challenges to social development and affects people's physical and mental health globally (2). Various negative emotions and psychological problems caused by the COVID-19 crisis cannot be ignored. Due to the uncertainty and fear associated with the pandemic, several studies have predicted an increasing trend in suicide during the outbreak of COVID-19 (3–5). Different studies have also reported that the pandemic has led to mental health problems in a variety of populations such as infected patients, quarantined populations and medical staff (6–10).

Medical workers play an important role in helping infected people, reducing deaths and injuries caused by epidemics, and controlling the progress of epidemics (11). Whenever the epidemic breaks out, a large number of medical workers are involved in the prevention and treatment of coronavirus infection, facing strong work intensity and a great risk of occupational exposure. Such working status has a huge adverse impact on the mental health status of them. Previous studies have already shown that healthcare workers suffered from anxiety, depression, burnout, insomnia, and post-traumatic stress disorder during epidemic (9, 12–14). As far as we know, the occurrence of psychological problems may negatively affect the attention, cognitive function, and clinical decision making of medical staff, leading to an increased incidence of medical errors and accidents, and posing a risk to patients (15). Therefore, the psychological status of medical staff needs to be paid attention to. Currently, there is limited guidance on how best to support healthcare workers in responding to public health emergencies, such as pandemics. This study aimed to figure out the psychological status of medical staff during the COVID-19 pandemic, analyze potential influencing factors, and provide references for further measures to address their psychological problems.

### 1.2 Research hypotheses

Based on a comprehensive review of the literature on healthcare workers' psychological responses during infectious disease outbreaks, as well as the specific context of mobile cabin hospitals during the COVID-19 pandemic, the following hypotheses were formulated:

Exposure-Related Hypothesis: Medical staff working in high-exposure departments (i.e., the confirmation department, which treats confirmed COVID-19 cases) would report a significantly higher prevalence and severity of psychological distress (GHQ-12 score ≥4) compared to those in lower-exposure departments (administration and logistics).

Prior Mental Health Hypothesis: A previous history of anxiety or depression would be a significant and independent risk factor for experiencing current emotional distress during the outbreak, representing one of the strongest predictors of both the presence (GHQ-12 ≥4) and severity (GHQ-12 total score) of psychological problems.

Demographic Vulnerability Hypothesis: Specific demographic subgroups, including female healthcare workers, those who are married without children, and individuals aged 30–39 years, would exhibit higher rates of psychological distress due to the compounded stress of work and family responsibilities during the epidemic.

Occupational and Gender-Specific Symptom Hypothesis: The psychological impact of the epidemic would manifest differently across occupations and genders. Specifically, we hypothesized that: (a) doctors would report a greater loss of self-confidence and self-worth compared to nurses, reflecting higher decision-making pressure; and (b) female staff would report higher rates of insomnia related to worry compared to their male counterparts.

Cumulative Stress Hypothesis: A greater number of deployments to mobile cabin hospitals (≥2 times) and a longer duration of the current support assignment (≥1 month) would be positively associated with the severity of psychological distress, reflecting a cumulative effect of prolonged exposure to a high-stress, high-risk environment.

## 2 Methods

### 2.1 Participants

A questionnaire survey was conducted from December 9, 2022, to December 11, 2022. The study selected participants based on convenience sampling. The target population was medical staff from mobile cabin hospitals in Beijing, China. A total of 266 people voluntarily took part in. The participants consisted of three occupational groups: doctors, nurses, and others (pharmacists, medical imaging technologists, medical officers, cleaners, security guards, etc.), and three departments: the confirmation department (which treated confirmed cases), the administration department, and the logistics department. We distributed the questionnaire online that could enable all the medical staff could access to it. Medical staff who volunteered to participate in were asked to complete the questionnaire independently and submitted online. The questionnaire detailed the purpose of the survey, the content, the collection of information and the way of using the results. All questions had to be answered to ensure the integrity of the results.

### 2.2 Instrument

The questionnaire consisted of a general demographic characteristics questionnaire and the 12-item General Health Questionnaire (GHQ-12) (16). The former part mainly includes the gender, age, marital status, occupation, the number of times, duration and position of the supporting mobile cabin hospital, and whether they have suffered anxiety and depression in the past.

The GHQ is one of the most popular and widely used screening instruments for detecting mental health (17). The GHQ-12 was selected for this study due to the busy work of the medical staff at mobile cabin hospitals. The reliability and validity of the Chinese version of the GHQ-12 have been examined by Chinese scholars, and Professor Yang confirmed its good reliability and validity in screening for psychological disorders by testing the suitability of the questionnaire in mainland China with appropriate language adjustments (18).

The questionnaire consists of 12 questions that evaluate one's psychological distress or general wellbeing with four possible answers: less than usual, same as usual, more than usual, and much more than usual. A bimodal rating scale (0-0-1-1) was used with a total score range of 0 to 12. The GHQ-12 includes six positive subscales and six negative subscales. A score of ≥4 is considered an indicator of declining mental health. The higher the score, the more severe the psychological distress.

### 2.3 Statistical analysis

Data were analyzed using SPSS 26.0 and R 4.2.0. Categorical variables were presented as frequencies and composition ratios, while continuous variables were described as mean ± standard deviation or median (interquartile range). In univariate analysis, comparisons between groups were performed using the chi-square test or Fisher's exact test for categorical variables, and the Mann-Whitney *U* test or Kruskal-Wallis *H* test for continuous variables. The significance level was set at α = 0.05 (two-tailed).

To further control for confounding factors and quantify the impact of various factors on psychological status, these regression analyses were conducted: Negative Binomial Regression: Given that the GHQ-12 total score is a count variable exhibiting overdispersion (variance substantially exceeding the mean), negative binomial regression was employed to analyze its influencing factors. The Akaike Information Criterion (AIC) and Bayesian Information Criterion (BIC) were compared among Poisson, negative binomial, and Gaussian regression models, confirming that the negative binomial model provided the best fit. Results were presented as incidence rate ratios (IRR) with 95% confidence intervals (CI). Binary Logistic Regression: The presence of emotional distress (GHQ-12 total score ≥4) was set as the dependent variable. Variables (gender, age, marital status, occupation, department, number of support deployments, duration of current support, and previous history of anxiety/depression), were entered as independent variables to construct a multivariate model. Variance inflation factor (VIF) was calculated to diagnose multicollinearity, with VIF < 5 indicating no severe multicollinearity. Results were presented as adjusted odds ratios (aOR) with 95% CI. Multicollinearity was also assessed using the correlation matrix (VIF >10 or correlation coefficient >0.7 indicated the presence of multicollinearity).

### 2.4 Ethics approval

It was determined that this study was exempt from ethical approval by the Medical Ethics Committee of Beijing Hospital of Traditional Chinese Medicine, since it posed low risk and did not involve any intervention with patients. This study was conducted in accordance with the Declaration of Helsinki.

## 3 Results

The demographic characteristics of the participants are presented in Table 1. A total of 266 medical staff were surveyed, of whom the ratio of men to women was approximately one to four. Marital status was divided into three categories, with 47.7% single and 41.4% married with children, and the proportion of married without children workers was 10.9%. The highest percentages of interviewees were nurses (69.9%). There were 223 medical workers working in the confirmation department, accounting for 83.8%. The majority of medical staff was under 40 years old. During the pandemic, 242 medical staff (91.0%) supported the mobile cabin hospital once, 19 (7.1%) supported it twice, and 5 (1.9%) supported it three times or more. The number of medical workers who had been in the mobile cabin hospital for 1 to 2 weeks was 5.3%; having been there for 3 to 4 weeks accounted for 44.4%; only 10.5% of medical staff had been there for 1 month or longer; and the length of support for 39.8% was undetermined. 18.8% of medical staff had previous anxiety and depression.

**Table 1:** Demographic characteristics of 266 medical staff associated with emotional distress and GHQ-12 total score.

Variables	Total, *n* (%)	Declining mental health, *n* (%)	No emotional distress, *n* (%)	P1	GHQ-12 total score mean (SD)	P2
Sex				0.575^a^		0.526^b^
Male	59 (22.2)	16 (27.1)	43 (72.9)		2.93 (3.248)	
Female	207 (77.8)	64 (30.9)	143 (69.1)		2.70 (3.029)	
Marital status				0.851^a^		0.251^d^
Single	127 (47.7)	37 (29.1)	90 (70.9)		2.48 (2.878)	
Married with children	29 (10.9)	10 (34.5)	19 (65.5)		3.55 (3.851)	
Married without children	110 (41.4)	33 (30.0)	77 (70.0)		2.85 (3.054)	
Occupation				0.36^a^		0.42^d^
Doctor	56 (21.1)	18 (32.1)	38 (67.9)		3.30 (3.687)	
Nurse	186 (69.9)	52 (28.0)	134 (72.0)		2.52 (2.823)	
Others	24 (9.0)	10 (41.7)	14 (58.3)		3.25 (3.287)	
Age, years				0.428^c^		0.23^d^
< 30	122 (45.9)	35 (28.7)	87 (71.3)		2.59 (3.193)	
30–39	101 (38.0)	34 (33.7)	67 (66.3)		3.13 (3.173)	
40–49	28 (10.5)	9 (32.1)	19 (67.9)		2.46 (2.769)	
50–59	15 (5.6)	2 (13.3)	13 (86.7)		1.87 (1.302)	
Current work department				0.198^a^		0.199^d^
Confirmation department	223 (83.8)	72 (32.3)	151 (67.7)		2.85 (3.091)	
Administration department	31 (11.7)	6 (19.4)	25 (80.6)		2.48 (3.129)	
Logistics department	12 (4.5)	2 (16.7)	10 (83.3)		1.58 (2.503)	
Times of supporting the mobile cabin hospital				0.876^c^		0.277^d^
1	242 (91.0)	72 (29.8)	170 (70.2)		2.64 (2.971)	
2	19 (7.1)	6 (31.6)	13 (68.4)		4.16 (4.153)	
3 times or more	5 (1.9)	2 (40.0)	3 (60.0)		2.40 (2.510)	
The duration of this support				0.892^c^		0.988^d^
1–2weeks	14 (5.3)	3 (21.4)	11 (78.6)		2.14 (1.994)	
3–4weeks	118 (44.4)	35 (29.7)	83 (70.3)		2.76 (3.034)	
1 month or longer	28 (10.5)	9 (32.1)	19 (67.9)		2.86 (3.341)	
Uncertain	106 (39.8)	33 (31.1)	73 (68.9)		2.78 (3.189)	
With previous anxiety and depression				0^a^		0^b^
Yes	50 (18.8)	35 (70.0)	13 (30.0)		5.24 (3.532)	
No	216 (81.2)	45 (20.8)	171 (79.2)		2.17 (2.651)	

^a^Categorical data were detected by chi-square test; ^b^Continuous data were analyzed using Mann-Whitney *U* test; ^c^Categorical data were detected by Kruskal–Wallis *H*-test; ^d^Continuous data were analyzed using Kruskal–Wallis *H*-test. GHQ-12, 12-item General Health Questionnaire; SD, standard deviation.

Negative binomial regression analysis was performed to identify factors associated with the GHQ-12 total score in Table 2. The results showed that a previous history of anxiety or depression was the strongest predictor of psychological distress, with those having such a history exhibiting a GHQ-12 score 2.57 times higher than those without (IRR = 2.57, 95% CI: 1.76–3.75, *P* < 0.001). Regarding age, using the 50–59 age group as the reference, the 30–39 age group had a significantly higher GHQ-12 score (IRR = 2.18, 95% CI: 1.05–4.56, *P* = 0.037); the < 30 age group also showed a trend toward higher scores, though it did not reach statistical significance (IRR = 2.15, 95% CI: 0.95–4.85, *P* = 0.067). In addition, using the logistics department as the reference, staff in the confirmation department had a higher level of psychological distress that approached significance (IRR = 2.06, 95% CI: 0.90–4.71, *P* = 0.086). Compared to those with three or more support deployments, staff with two deployments also showed a trend toward higher GHQ-12 scores (IRR = 2.98, 95% CI: 0.86–10.33, *P* = 0.085). No significant associations were found for sex, marital status, occupation, or duration of support (all *P* > 0.05).

**Table 2:** Association between GHQ-12 score and demographic characteristics of 266 medical staff.

Variables	SE	IRR	95% Cl	*P*
Sex				
Male	0.218	1.074	0.701–1.647	0.742
Female		1		
Marital status				
Single	0.2327	0.711	0.45–1.122	0.142
Married with children	0.2846	0.769	0.44–1.343	0.355
Married without children		1		
Occupation				
Doctor	0.3317	0.621	0.324–1.19	0.151
Nurse	0.3065	0.639	0.35–1.165	0.144
Others		1		
Age, years				
< 30	0.4164	2.145	0.948–4.85	0.067
30–39	0.3753	2.183	1.046–4.555	**0.037**
40–49	0.4057	1.514	0.683–3.352	0.307
50–59		1		
Current work department				
Confirmation department	0.4215	2.062	0.902–4.71	0.086
Administration department	0.4641	1.668	0.672–4.143	0.27
Logistics department		1		
Times of supporting the mobile cabin hospital				
1	0.5731	2.177	0.708–6.695	0.175
2	0.634	2.982	0.861–10.332	0.085
3 times or more		1		
The duration of this support				
1–2weeks	0.3635	0.711	0.349–1.45	0.349
3–4weeks	0.1683	0.957	0.688–1.331	0.796
1 month or longer	0.2716	1.071	0.629–1.825	0.799
Uncertain		1		
With previous anxiety and depression				
Yes	0.1924	2.57	1.763–3.748	**0**
No		1		

Of the 266 medical staff who participated in this survey, 80 had a decline in mental health, accounting for about one-third of the sample size. The highest percentages in their categories were women (30.9%), married without children (34.5%), staff in other occupations (41.7%), and those aged 30–39 years (33.7%). The proportion of medical staff who worked in the confirmation department (32.3%) with a decline in mental health was significantly higher than those in other two departments. The proportion of people who had supported mobile cabin hospitals three times or more (40.0%) and stayed there for more than 1 month (32.1%) with emotional distress was higher. There was no significant difference in the proportion of emotional distress in the total sample among the above categories. In contrast, 70% of those with previous anxiety and depression states reported the presence of emotional distress in this survey. Only 20.8% of those without previous anxiety or depression states had emotional distress recently. There is a statistically significant difference between healthcare workers with previous anxiety or depression states and those without in terms of a decline in mental health.

The differences in the occupational variables on GHQ-12 item 4 (*P* = 0.001), item 6 (*P* = 0.008), item 10 (*P* = 0.033) and item 11 (*P* = 0.007) were statistically significant (Table 3). Among the department variables only item 2 (*P* = 0.016) had a significant difference (Table 4). Significant differences were found in GHQ-12 item 2 (*P* = 0.038), item 7 (*P* = 0.029), and item 11 (*P* = 0.011) of the gender variables (Table 5). To further investigate the factors influencing the GHQ-12 total score, multiple linear regression analysis was performed (Table 6). Multifactorial binary logistic regression analysis was used. The results showed that the previous presence of anxiety and depressive states was a risk factor for the recent emotional distress among the medical staff in the mobile cabin hospitals (Table 7).

**Table 3:** Association between GHQ-12 items and occupation.

GHQ-12 items	Doctors, *n* (%)	Nurses, *n* (%)	Others, *n* (%)	*P*
1. Able to concentrate	47 (83.9)	161 (86.6)	22 (91.7)	0.674
2. Too worry to sleep	17 (30.4)	76 (40.9)	10 (41.7)	0.350
3. Playing a useful part	46 (82.1)	168 (90.3)	20 (83.3)	0.177
4. Capable of making decisions	47 (83.9)	176 (94.6)	18 (75.0)	0.001
5. Under stress	30 (53.6)	90 (48.4)	13 (54.2)	0.724
6. Could not overcome difficulties	14 (25.0)	17 (9.1)	4 (16.7)	0.008
7. Enjoy your routine activities	28 (50.0)	116 (62.4)	15 (62.5)	0.244
8. Face up to problems	46 (82.1)	163 (87.6)	19 (79.2)	0.335
9. Feeling unhappy and depressed	19 (33.9)	53 (28.5)	7 (29.2)	0.736
10. Losing confidence	8 (14.3)	19 (10.2)	7 (29.2)	0.033
11. Thinking of self as worthless	10 (17.9)	9 (4.8)	2 (8.3)	0.007
12. Feeling reasonably happy	35 (62.5)	128 (68.8)	15 (62.5)	0.604

Data were examined using chi-square test and Fisher Exact test. GHQ-12, 12-item General Health Questionnaire.

**Table 4:** Association between GHQ-12 items and department.

GHQ-12 items	Confirmation department, *n* (%)	Administration department, *n* (%)	Logistics department, *n* (%)	*P*
1. Able to concentrate	190 (85.2)	28 (90.3)	12 (100.0)	0.344
2. Too worry to sleep	94 (42.2)	8 (25.8)	1 (8.3)	0.016
3. Playing a useful part	194 (87.0)	28 (90.3)	12 (100.0)	0.514
4. Capable of making decisions	204 (91.5)	26 (83.9)	11 (91.7)	0.298
5. Under stress	113 (50.7)	16 (51.6)	4 (33.3)	0.495
6. Could not overcome difficulties	30 (13.5)	5 (16.1)	0 (0.0)	0.463
7. Enjoy your routine activities	131 (58.7)	19 (61.3)	9 (75.0)	0.558
8. Face up to problems	190 (85.2)	28 (90.3)	10 (83.3)	0.694
9. Feeling unhappy and depressed	71 (31.8)	6 (19.4)	2 (16.7)	0.247
10. Losing confidence	28 (12.6)	4 (12.9)	2 (16.7)	0.852
11. Thinking of self as worthless	16 (7.2)	3 (9.7)	2 (16.7)	0.302
12. Feeling reasonably happy	146 (65.5)	22 (71.0)	10 (83.3)	0.469

Data were examined using chi-square test and Fisher Exact test. GHQ-12, 12-item general health questionnaire.

**Table 5:** Association between GHQ-12 items and gender differences.

GHQ-12 items	Male, *n* (%)	Female, *n* (%)	*P*
1. Able to concentrate	52 (88.1)	178 (86.0)	0.671
2. Too worry to sleep	16 (27.1)	87 (42.0)	0.038
3. Playing a useful part	53 (89.8)	181 (87.4)	0.618
4. Capable of making decisions	52 (88.1)	189 (91.3)	0.462
5. Under stress	30 (50.8)	103 (49.8)	0.883
6. Could not overcome difficulties	11 (18.6)	24 (11.6)	0.158
7. Enjoy your routine activities	28 (47.5)	131 (63.3)	0.029
8. Face up to problems	52 (88.1)	176 (85.0)	0.547
9. Feeling unhappy and depressed	18 (30.5)	61 (29.5)	0.877
10. Losing confidence	10 (16.9)	24 (11.6)	0.277
11. Thinking of self as worthless	10 (16.9)	11 (5.3)	0.011
12. Feeling reasonably happy	39 (66.1)	139 (67.1)	0.880

Data were examined using chi-square test and Fisher Exact test. GHQ-12, 12-item General Health Questionnaire.

**Table 6:** Multiple linear regression analysis of factors associated with GHQ-12 total score.

Variables	B	SE	β	*t*	*p*	Tolerance	VIF
Constant term	1.175	0.226		5.194	0		
Sex	−0.033	0.066	−0.03	−0.498	0.619	0.882	1.133
Marital status	0.056	0.039	0.114	1.413	0.159	0.48	2.082
Occupation	0.065	0.053	0.075	1.22	0.224	0.822	1.217
Age	−0.061	0.043	−0.114	−1.441	0.151	0.497	2.013
Current work department	−0.081	0.054	−0.09	−1.496	0.136	0.874	1.144
Times of supporting the mobile cabin	−0.005	0.071	−0.004	−0.072	0.942	0.971	1.03
The duration of this support	0.026	0.026	0.057	1	0.318	0.961	1.041
With previous anxiety and depression	−0.5	0.067	−0.426	−7.503	0	0.971	1.03
*R* ^2^	0.319						
Adjusted *R*^2^	0.317						
F	F = 7.769, *p* = 0.000						
D-W	1.865						

**Table 7:** Multivariate binary logistic regression analysis of factors associated with emotional distress (GHQ-12 ≥4) among medical staff.

Variables	Regression coefficient	SE	Wald *X*^2^	*P*	OR (95% CI)
Sex					
Male vs. Female	−0.094	0.422	0.05	0.823	0.91 (0.398, 2.082)
Marital status					
Single vs. Married with children	−0.452	0.499	0.821	0.365	0.636 (0.239, 1.692)
Single vs. Married without children	−0.67	0.598	1.255	0.263	0.512 (0.159, 1.652)
Occupation					
Doctor vs. NURSE	−1.521	0.722	4.434	0.035	0.219 (0.053,0.9)
Doctor vs. Others	−1.674	0.665	6.333	0.012	0.187 (0.051,0.691)
Age, years					
< 30 vs. 30–39	2.429	1.048	5.373	0.02	11.346 (1.455,88.459)
< 30 vs. 40–49	2.343	0.963	5.92	0.015	10.416 (1.577,68.788)
< 30 vs. 50–59	2.323	1.006	5.335	0.021	10.202 (1.422,73.217)
Current work department					
Confirmation department vs. Administration department	0.826	0.905	0.833	0.361	2.283 (0.388,13.448)
Confirmation department vs. Logistics department	−0.613	1.046	0.343	0.558	0.542 (0.07,4.209)
Times of supporting the mobile cabin hospital					
1 vs. 2	0.636	1.317	0.234	0.629	1.89 (0.143,24.961)
1 vs. 3 times or more	0.492	1.454	0.114	0.735	1.635 (0.095,28.278)
The duration of this support					
1–2 weeks vs. 3–4 weeks	−1.295	0.834	3.309	0.121	0.274 (0.053,1.405)
1–2 weeks vs. 1 month or longer	−0.039	0.345	0.013	0.91	0.962 (0.489,1.889)
1–2 weeks vs. uncertain	0.382	0.543	0.497	0.481	1.466 (0.506,4.245)
With previous anxiety and depression					
Yes vs. no	2.583	0.423	37.285	0	13.236 (5.777,30.326)

## 4 Discussion

Our study investigated the psychological status of medical staff in hospitals in Beijing during the COVID-19 pandemic, aiming to explore the psychological impact of epidemic on medical staff. As we know, the general public has to face recurrent small-intensity outbreaks and constant mutations of the virus (19), meanwhile the healthcare workers need to face enormous work and mental stress (20–23). Under permanent epidemic prevention and control, healthcare workers face abrupt workload, variable task lengths, unknown working environments, and the risk of infection (24). Continuous tension and overload of work make them physically and psychologically exhausted. Therefore, we should focus not only on their physical state but also on their mental health.

In our survey, 30.1% of healthcare workers have experienced a decline in their mental health recently. The higher prevalence of emotional distress was found among women, married without children and aged 30–39 years. In the same stressful environment, a greater proportion of men felt they had lost their self-worth compared to women. Such people tend to bear more stress in their families and society. So some personal characteristics can be found to increase the risk of mental health problems in healthcare workers (25). The previous psychological state of medical staff also affects their psychology in the face of the epidemic. Having a previous state of anxiety and depression was the risk factor for healthcare workers to suffer emotional distress. In addition, women showed higher rates of insomnia due to worry compared to men. There is a facilitative relationship between insomnia symptoms and psychological problems (26). People with recurrent insomnia are more likely to develop psychological problems, and psychological problems also promote the occurrence of insomnia (27, 28). Insomnia among healthcare workers has proven to be common during COVID-19 (29). Therefore, it is equally important to focus on the quality of sleep in healthcare workers, as good sleep can promote the effective deployment of individuals' cognition-related resources and increase the likelihood of utilizing emotion regulation strategies to regulate emotional experiences (30).

Also, medical staff who worked in the confirmation department had a higher incidence of a decline in mental health. Those supporting mobile cabin hospitals 3 times or more and duration of this support for 1 month or longer were more likely to develop emotional distress. And having a previous state of anxiety and depression was the risk factor for healthcare workers to suffer emotional distress. The degree of decline in mental health was significantly lower among those without previous anxiety or depression than among those with.

Participating in the front-line treatment of COVID-19 patients makes medical workers more prone to psychological problems. Those working as doctors and other professions, working in the confirmation department, supporting mobile cabin hospitals 3 times or more and duration of this support for 1 month or longer were more likely to develop emotional distress. A survey conducted by our team in March 2020 on the incidence of emotional distress among medical staff showed that the percentage of medical staff experiencing emotional distress was about 16.7% (31), much lower than the 30.1% obtained in this survey. Wang H J et al. (32) found that 20% of the healthcare workers (*n*= 104,980) in Guangdong, China experienced moderate to severe anxiety and 39.4% suffered from depression, and healthcare workers who had potential or direct contact with infected patients were more likely to fall into stress states and had higher severity of insomnia. Their emotional distress may be related to the recurring outbreaks of the pandemic. The most medical staff has to throw themselves into persistent prevention and control tasks. The continuous intensive work and high exposure risk tend to cause repeated psychological trauma to medical staff.

The outbreak of epidemic diseases make medical staff work under huge pressure and mental stress. Therefore, the prevention and intervention measures should be conducted to improve the mental health level of them. Training of medical staff, planning and allocation of staff, provision of adequate protective equipment and establishment of a mental health team for professionals are important options (33). Moreover, attention should be paid to screening and providing targeted help to healthcare workers in need during epidemics. According to our study: female, married without children and aged 30–39 years, having a previous state of anxiety and depression, and are at higher risk of exposure may be more in need of help. For those reporting mental health problems, a three-stage stepped intervention including a psychological first aid workshop, a psychoeducational workshop, and a brief CBT group program may be helpful. To increase accessibility, this intervention may be delivered by general medical professionals (peers) trained by mental health professionals (34).

However, this survey still has certain limitations. Firstly, the sample size of the survey respondents is not large enough, which may lead to bias in the results. Secondly, convenience sampling was used in this survey. Because the sample representativeness is influenced by chance factors, it is not suitable to generalize the study results to whole population. Thirdly, due to time constraints, the questionnaire was designed relatively simple in order to reduce the burden on medical staff, yet richer questionnaire content would help improve reliability and structural validity. Fourthly, the cross-sectional design precludes any causal inferences; the associations identified in this study should be interpreted as correlational rather than causal. Longitudinal studies are needed to elucidate the temporal relationships between exposure factors (e.g., work duration, department) and the development of psychological distress. Lastly, due to the rapidly changing nature of the COVID-19 pandemic and national policies, the data collected over a short period (3 days) may not reflect the dynamic psychological status of healthcare workers across different phases of the outbreak. Future research should consider repeated assessments over time to capture these fluctuations.

## 5 Conclusion

In conclusion, the outbreak of epidemic make medical staff face a higher risk of psychological problems. Grasping this mental health impact will make policy makers and governance aware of the importance of considering the mental health needs of healthcare workers in the preparation for, during, and after such outbreaks.

## Data Availability

The original contributions presented in the study are included in the article/supplementary material, further inquiries can be directed to the corresponding author.
